# Editorial: The ever so elusive pathogen-harboring biofilms on abiotic surfaces in the food and clinical sectors: the good, the bad and the slimy

**DOI:** 10.3389/fcimb.2024.1374693

**Published:** 2024-02-09

**Authors:** Olivier Habimana, Arnaud Bridier, Efstathios Giaouris

**Affiliations:** ^1^ Department of Biotechnology and Food Engineering, Guangdong Technion-Israel Institute of Technology, Shantou, China; ^2^ Antibiotics, Biocides, Residues and Resistance Unit, Fougeres Laboratory, French Agency for Food, Environmental and Occupational Health & Safety (ANSES), Fougeres, France; ^3^ Laboratory of Food Microbiology and Hygiene, Department of Food Science and Nutrition, School of the Environment, University of the Aegean, Myrina, Lemnos, Greece

**Keywords:** biofilms, food safety, bacterial pathogen, infectious diseases, stress adaptation, multicellularity, public health, antimicrobial resistance (AMR)

Biofilms are communities of microorganisms adhered to surfaces. These are enclosed in sticky substances called extracellular polymeric substances (EPS), forming higher multicellular structures that allow microorganisms to resist adverse environmental conditions, such as nutrient absence, drought, pH extremes, host immune responses, and many other antimicrobial interventions (Ciofu et al., 2022; Pai et al., 2023). Biofilms also containing pathogenic microorganisms can develop on a wide range of abiotic surfaces, such as those encountered in food processing and medical sectors, allowing the enclosed microorganisms to persist even after regular cleaning and disinfection procedures, possibly leading to cross-contamination of foods, foodborne disease outbreaks and hard to treat infections (Charron et al., 2023). As editors of this Research Topic on pathogen-harboring biofilm on abiotic surfaces in the food and clinical sectors, we were delighted to receive and review some fascinating research articles within this field. This editorial briefly reports the main findings, conclusions, and perspectives of each accepted article.

Dairy processing plants offer an ideal environment for biofilm development due to milk residue enriched in carbohydrates, proteins, and fats (Yuan et al., 2023). Of the many biofilm-forming organisms that thrive in such environments, *Bacillus* spp. survive even after pasteurization due to their ability to be differentiated in heat resistant spores (Shemesh and Ostrov, 2020). Their presence is, therefore, of significant concern for the dairy industry because these bacteria can continuously contaminate food processing streams, ultimately affecting the safety of dairy products and causing their spoilage. The work carried out by Catania et al. demonstrated that *B. subtilis* and *B. cereus* isolates from processed cheese products that survived heat treatments could easily form biofilms on common food contact surfaces. While species-specific biofilm-forming variations were observed between the isolates, their biofilm-forming phenotype was linked to the presence of biofilm-related determinants in their genomes and their cell surface properties. Scanning electron microscopy (SEM) analysis revealed a complex biofilm structural architecture and an extracellular matrix covering and embedding bacterial cells. Given the fact that biofilm-forming *Bacillus* spp. organisms are more challenging to eradicate, also showing resistance to standard sanitization practices applied in dairy plants, their presence on dairy processing equipment surely poses a significant issue. Hence, it is crucial to expand our knowledge on biofilm formation by these and other heat-resistant microorganisms by investigating their molecular characteristics and biofilm-forming mechanisms under relevant food processing conditions. Such research could help devise new strategies for mitigating risk in food processing plants.

Multi-species biofilms cause problems in various environments, especially food processing ones. Compared to single-species biofilms, mixed-species ones are usually more resistant to various physicochemical stresses, including antimicrobials such as disinfectants (Kim et al., 2022). The knowledge of the microbiome composition and diversity in such biofilms found in food processing environments and their metabolic potential could assist in developing more powerful intervention techniques to combat pathogens in these environments. The research conducted by Palanisamy et al. aimed to determine biofilm composition, diversity, and functional potential in the beef processing industry. For this, the authors analyzed, through metagenomic sequencing, 75 biofilm drain samples collected from five different locations in three beef-processing plants at two different time points. Core microbiome analysis revealed that *Pseudomonas*, *Psychrobacter*, and *Acinetobacter* were the three most prevalent genera among the plants and locations, with a high microbiome diversity always present. Functional analysis unveiled the microbial communitys high metabolic potential with abundant genes related to metabolism, cell adhesion, motility, and quorum sensing (QS). Alarmingly, genes conferring resistance to quaternary ammonium compounds (QACs), which are common sanitizers in the food industry, were also detected. The presence of some other multi-functional genes and mobile genetic elements highlighted the dynamic nature of those microbial communities, which seem to be able to protect themselves against various environmental stresses through multiple defense mechanisms. Such studies, offering comprehensive snapshots of the microbial profiles of biofilms found in the food industry and elsewhere, contribute to gaining insights into the factors related to biofilm formation and persistence. This can ultimately help the development of more effective intervention strategies to control unwanted biofilm formation in many areas.

Nowadays, antimicrobial resistance (AMR) is one of the leading health issues humanity faces, with millions of lives being lost annually due to infectious diseases (de la Fuente-Nunez et al., 2023). Worldwide, methicillin-resistant *Staphylococcus aureus* (MRSA) is a serious and life-threatening bacterial pathogen presenting multidrug resistance (MDR) to the common beta-lactam antibiotics category and causing serious community, nosocomial, animal, and foodborne infections (Chalmers and Wylam, 2020). The problem has been recently further intensified with the emergence of vancomycin-resistant *S. aureus* (VRSA), given that vancomycin is still used as one of the first-line drugs for treating MRSA infections (Cong et al., 2019). Therefore, novel antimicrobials are urgently required to treat such severe infections efficiently. The work reported by Abd El-Hamid et al. aimed to develop and assess a novel nanocarrier system based on mesoporous silica nanoparticles (MPS-NPs) for free berberine (BR) as a plant-derived natural antimicrobial alkaloid against strong biofilm-producing and multi-virulent VRSA strains using *in vitro* and *in vivo* models. For this, the authors examined 95 *S. aureus* isolates from either milk samples collected from cows with mastitis or human pus samples. All isolates were confirmed as MRSA and they were resistant to both oxacillin and cefoxitin. Notably, vancomycin resistance was observed in 13.7% of these isolates, while 69.2% of VRSA isolates were also proven to be strong biofilm producers (*n* = 9). 44.4% of the latter isolates harbored all five virulence genes tested (*icaA*, *tst*, *clfA*, *hla*, and *pvl*), and 88.9% were also multi-virulent. The BR-loaded MPS-NPs were found to present excellent *in vitro* antimicrobial and antibiofilm properties. Significant downregulation of virulence and *agr* genes was also displayed in all strong biofilm-producing and multi-virulent VRSA strains following their exposure to BR-loaded MPS-NPs in both *in vitro* and *in vivo* mice models. In addition, the treatment of VRSA-infected mice with BR-loaded MPS-NPs attenuated the upregulated expression of pro-inflammatory cytokines genes and, in parallel, significantly reduced apoptosis via downregulation of pro-apoptotic genes. Overall, the results of this study seem important since they advocate for the promising application of BR-loaded MPS-NPs as an efficient therapeutic alternative for controlling multi-virulent VRSA strains. Such studies could help tackle AMR’s nightmare problem early and effectively.

While earlier work on antibiofilm strategies has primarily focused on inhibiting biofilm formation, more recent studies have explored the mechanisms of bacterial dispersion from biofilms as a potential target for antimicrobial design by focusing on inducing sessile cells back into planktonic states, thereby causing the cells to become again more susceptible to antimicrobial exposure (Wille and Coenye, 2020). The work conducted by Wang et al. studied biofilm dispersal in *Pseudomonas aeruginosa*, a significant biofilm-forming pathogen responsible for acute and chronic infections in humans and animals. Various strategies have been employed to counteract *P. aeruginosa* biofilms, including QS regulators, bioactive molecules, bacteriophages, antimicrobial peptides (AMPs), and plant extracts. AMPs are increasingly viewed as potential antibiotic alternatives due to rising antibiotic resistance. Although research on biofilm dispersion induced by AMPs is still limited, several AMPs have demonstrated efficacy in inhibiting biofilm formation and eliminating established biofilms. Based on the authors previous study showing the dispersal efficacy of a mouse antimicrobial peptide CRAMP-34 using multi-omics approaches, their most recent work scrutinized the extracellular metabolic profiles of *P. aeruginosa* biofilms following CRAMP-34 exposure to further elucidate its biofilm-dispersing mechanism. Their current results substantiated the biofilm-dispersing effect of CRAMP-34, marked by significant shifts in some extracellular metabolites such as glutamate, succinate, myoinositol, palmitic acid, and oleic acid. Moreover, combining CRAMP-34 with an antibiotic exhibited enhanced efficacy against dispersed bacteria and concurrently delayed the development of antibiotic resistance. These insights underscore the potential of leveraging CRAMP-34 as a biofilm dispersant, while its use in parallel with antibiotics may offer an innovative therapeutic strategy for mitigating biofilm-associated infections.

In the last years, an increasing interest has been focused on the potential impact of biocides, which are multi-targeted molecules that are daily and massively used in various environments, on cross-resistance to a variety of antimicrobials, including antibiotics (Maillard, 2018). Microorganisms mostly live within biofilms, and such collective and structured lifestyles greatly influence the way these adapt to stress. Therefore, understanding the potential interplay between biocide exposure, biofilm adaptation, and the emergence of AMR and cross-resistance is crucial to better controlling the burden of pathogenic microorganisms. The work conducted by Charron et al. showed that exposure to a biocidal active substance, the polyhexamethylene biguanide (PHMB), led to an increase in adaptive gentamicin cross-resistance in *Escherichia coli* biofilms. That adaptive cross-resistance was associated with an induction of gene expression associated with biofilm matrix production, stress responses, and membrane transport. This was also related to the modulation of biofilm architecture in response to biocide stress with the appearance of dense cellular clusters, altering gradients and microenvironmental conditions in the biological edifice and playing a role in the emergence of adaptive cross-resistance to the studied antibiotic. Overall, this work provided original data on the adaptive strategies of bacteria in biofilms exposed to biocidal stress and highlighted the potential side effects on AMR. Further, such intriguing studies should be dedicated to deeply understanding collective biofilm adaptation to biocides and antimicrobial cross-resistance emergence, focusing on various combinations of biocidal active substances and bacterial species.

In conclusion, articles published in this Research Topic “*The ever so elusive pathogen-harboring biofilms on abiotic surfaces in the food and clinical sectors: the good, the bad and the slimy”* emphasize the importance of biofilm collective behavior and ecological interactions in bacterial adaptive processes and survival to stress, including AMR. These also highlight some promising innovative anti-biofilm strategies to better control biofilm development by widespread bacterial pathogens. Future research on this topic will hopefully allow us to delve even deeper into the functioning of biofilms and better conceive the positive and negative properties of these complex biological structures, progressing always for the benefit of the health of humans, animals, and the environment.

## Author contributions

OH: Writing – original draft, Writing – review & editing. AB: Writing – original draft, Writing – review & editing. EG: Writing – original draft, Writing – review & editing.
